# Consequences of Low Dose Ionizing Radiation Exposure on the Hippocampal Microenvironment

**DOI:** 10.1371/journal.pone.0128316

**Published:** 2015-06-04

**Authors:** Munjal M. Acharya, Neal H. Patel, Brianna M. Craver, Katherine K. Tran, Erich Giedzinski, Bertrand P. Tseng, Vipan K. Parihar, Charles L. Limoli

**Affiliations:** Department of Radiation Oncology, University of California Irvine, Irvine, CA, 92697–2695, United States of America; ENEA, ITALY

## Abstract

The response of the brain to irradiation is complex, involving a multitude of stress inducible pathways that regulate neurotransmission within a dynamic microenvironment. While significant past work has detailed the consequences of CNS radiotherapy following relatively high doses (≥ 45 Gy), few studies have been conducted at much lower doses (≤ 2 Gy), where the response of the CNS (like many other tissues) may differ substantially from that expected from linear extrapolations of high dose data. Low dose exposure could elicit radioadaptive modulation of critical CNS processes such as neurogenesis, that provide cellular input into hippocampal circuits known to impact learning and memory. Here we show that mice deficient for chemokine signaling through genetic disruption of the CCR2 receptor exhibit a neuroprotective phenotype. Compared to wild type (WT) animals, CCR2 deficiency spared reductions in hippocampal neural progenitor cell survival and stabilized neurogenesis following exposure to low dose irradiation. While radiation-induced changes in microglia levels were not found in WT or CCR2 deficient animals, the number of Iba1+ cells did differ between each genotype at the higher dosing paradigms, suggesting that blockade of this signaling axis could moderate the neuroinflammatory response. Interestingly, changes in proinflammatory gene expression were limited in WT animals, while irradiation caused significant elevations in these markers that were attenuated significantly after radioadaptive dosing paradigms in CCR2 deficient mice. These data point to the importance of chemokine signaling under low dose paradigms, findings of potential significance to those exposed to ionizing radiation under a variety of occupational and/or medical scenarios.

## Introduction

The central nervous system (CNS) is sensitive to a variety of insults that include trauma, ischemia, depression, chemotherapy and ionizing radiation. Exposure to ionizing radiation can lead to a wide spectrum of radiolytic lesions throughout different cellular compartments that elicits a global stress response impacting metabolism, DNA repair, cell cycle progression, and survival [1–3]. Past data has found that irradiation also elicits a complex temporal response of secondary reactive processes involving oxidative stress and inflammation [4–7]. Common to most CNS insults however, is the marked sensitivity of the neurogenic process in the brain. While the rise and fall of neurogenesis in response to specific stimuli has been a topic of intense investigation, little doubt exists regarding the capability of relatively higher doses (≥ 2 Gy) of ionizing radiation to inhibit this process [2,3,6]. Radiation-induced inhibition of neurogenesis is dose-dependent and persistent, and after clinically relevant dosing paradigms (≥ 45 Gy), is likely to contribute significantly to the onset and progression of cognitive deficits. Radiation inhibits neurogenesis via the depletion of radiosensitive populations of neural stem and progenitor cells (NSCs) residing in the sub-granular zone (SGZ) of the dentate gyrus (DG), thereby blocking the addition of new cells in the brain that contribute to hippocampal-dependent learning and memory. Other mechanisms regulate the inhibition and/or recovery of neurogenesis and include a variety of stress responsive of signaling mechanisms that impact the level of neuroinflammation [5, 8, 9].

Less is known however, regarding the response of the CNS to low dose ionizing radiation (LDIR, ≤ 2 Gy), typically encountered from environmental or occupational exposures, or from various medical procedures. Lower dose exposures have also been found to significantly perturb metabolism leading to elevated reactive oxygen (ROS) and nitrogen (RNS) species, that can trigger changes in the redox balance of the CNS microenvironment [2, 10]. Many of these alterations may elicit opposing or compensatory effects in the CNS that exhibit trends opposite to that observed after higher dose exposures. Precisely how LDIR impacts the CNS to compromise or facilitate functionality was a focus of the current investigation.

Our past data has shown that low dose exposure (≤ 1 Gy) to heavy ions was associated with varying degrees of cognitive dysfunction, suggesting that certain radiation types pose potential risks to the long-term cognition [3]. At similar low dose ranges, protons were found to elicit similar behavioral decrements and a persistent reduction in dendritic complexity and spine density, linking certain behavioral deficits to structural alterations of neurons throughout the hippocampus [11, 12]. While studies with charged particles have pointed to potential detrimental effects in the CNS, few studies have addressed the importance of inflammatory signaling in the brain subjected to low and/or adaptive doses of photon radiation.

A wealth of data has substantiated the importance of neuroinflammation in a wide range of CNS pathologies [13]. Chemokine receptors and ligands are important mediators of acute and chronic neuroinflammation that regulate immune cell responses following CNS injury [14]. Microglia, the resident immune cell of the CNS, express chemokine receptors, and the chemokine (C-C motif) receptor 2 (CCR2) has been shown to play an important role in modulating inflammatory responses in the irradiated CNS [8, 15–17]. CCR2 expression is also found on hippocampal NSCs, granule cell neurons in the DG, and pyramidal cell neurons in the CA1 [19,20]. Past work measuring a number of CNS endpoints have suggested that CCR2 knockout (CCR2-KO) mice were protected against radiation alone or in combination with traumatic brain injury [15]. Compared to wild type (WT) mice, CCR-deficient animals showed reduced neuroinflammation, increased neurogenesis, and improved hippocampal based learning and memory [8, 15].

The foregoing data points to the important role of CCR2 mediated inflammatory signaling in the CNS following higher doses (4 – 10 Gy) of acute cranial irradiation. To further understand the impact of LDIR on the response of the CNS, we utilized WT and CCR2-KO mice to study hippocampal neurogenesis, along with cellular and gene expression alterations in inflammatory signaling following acute low dose and radioadaptive dosing paradigms.

## Materials and Methods

### Animals, Radiation Exposure and BrdU treatment

**Fig 1:**
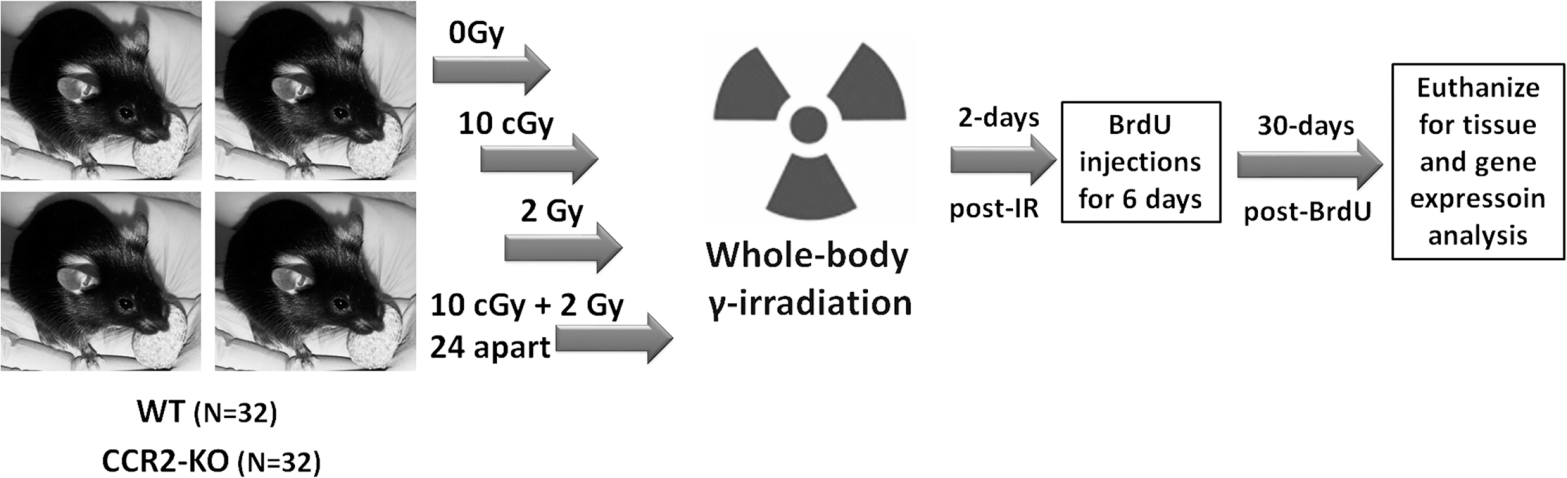
Schematics of research design. WT and CCR2-KO mice were divided into 4 experimental groups (0, 10 cGy, 2Gy, 10 cGy + 2 Gy 24h apart). Mice were restrained briefly in plexiglass chambers and given whole body gamma radiation using ^137^Cs irradiator at the dose rate of 1.07 Gy per minute. At 2 days post-irradiation, animals received 6 consecutive BrdU injections (50mg/kg, i.p.) and were euthanized 30 days later for immunohistochemical and qPCR analyses.

All animal procedures were carried out in compliance with the U.S. Department of Health and Human Services (DH&HS) Guide for the Care and Use of Laboratory Animals and this study was approved specifically by the University of California Institutional Animal Care and Use Committee (IACUC). Two months old, male transgenic CCR2-KO mice (stock 0004999) on a C57BL/6 background and WT C57BL/6 mice (stock 000664) were purchased from the Jackson Laboratories. Schematics of the research design are shown in **Fig 1**. A total of 64 mice were used in this study. A group of 32 mice per genetic background (WT or CCR2-KO) were divided into 4 groups: 0 Gy, 10 cGy, 2 Gy and 10 cGy + 2Gy. For radioadaptive dosing, the initial priming (10 cGy) and subsequent challenge (2 Gy) doses were separated by 24h. All irradiations were whole body and were conducted using ^137^Cs irradiator (J. L. Shepherd Associates, Glendale, CA, USA) at a dose rate of 1.07 Gy/min. Mice (n = 4 at a time) were briefly restrained in a compartmentalized Plexiglas container with an aeration space. Sham animals (0 Gy dose) were treated identically without exposure. To assess the impact of LDIR on hippocampal neurogenesis between transgenic CCR2-KO and WT mice, 5’-bromo-2’-deoxyuridine (BrdU; 50mg/kg i.p., Sigma-Aldrich, MO, USA) was injected for 6 consecutive days at 2 days post-irradiation. At 1-month post-irradiation, animals (n = 4 each group) were deeply anesthetized with isoflurane and euthanized via intracardiac perfusion using saline with heparin (10 U/ml, Sigma-Aldrich) followed by 4% paraformaldehyde (Acros Organics). Brains were cryoprotected in a serial gradient of buffered sucrose (10–30%, Sigma-Aldrich) and sectioned coronally (30μm thick) using a cryostat.

### Assessment of neurogenesis and microglia

To assess neurogenesis in the WT and CCR2-KO mice, free floating dual immunofluorescence staining procedure for BrdU and neuron-specific nuclear antigen (NeuN) was performed on representative sections from each experimental group as described previously [6, 20]. This technique provides for a relative quantification of neurogenesis, since unbiased stereology was not performed in the present study. To determine the status of resident microglia following irradiation, Iba-1 immunofluorescence staining was carried out using anti-Iba-1 antibody (1:500 dilution, Wako, [21]) and counterstained using Toto-3 iodide (Invitrogen, Carlsbad, CA) nuclear dye (1 μmol/L, 15 min). All immunofluorescence stained sections were imaged using laser-scanning confocal microscope (Nikon Eclipse TE2000-U, EZ-C1 interface, Japan) and 1-μm thick Z-stacks were obtained to visualize BrdU (red), NeuN (green), Iba-1 (green) and Toto-3 (pink) stained cells. A minimum of 50 BrdU-positive cells were counted for each animal, and the percentage of dual labeled BrdU-NeuN positive cells was derived from 4 individual animals in each group. Proliferating cells in the SGZ were quantified by counting the number of BrdU positive cells from 30 μm coronal sections (8 sections per animal) from each group. Similarly, Iba-1 positive resting microglia were quantified in each hippocampal subfield (DG, CA1 and CA3) from 30 μm thick sections (8 sections per animal) from each group.

### Gene expression analysis for inflammatory markers

To assess the impact of LDIR on inflammatory signatures within WT and CCR2-KO mice, quantitative real time PCR (qPCR) was performed to measure the expression of inflammatory markers (Iba-1, CD68 and CD11c) in the neocortex and hippocampus from each group (n = 4 per group). These markers were selected based on past experience and on published reports analyzing the impact of ionizing radiation on inflammatory signaling [8, 17]. Briefly, animals were deeply anesthetized with isoflurane and euthanized by decapitation. Brains were dissected using micro-scissors to separate hippocampal and cortical regions. Freshly dissected tissue samples were placed in RNAlater solution (Ambion, LifeTechnologies, CA, USA) and stored at -20°C. A maximum of 50mg of brain tissue was dissolved in RNA extraction buffer and Trizol (Ambion) for homogenization followed by treatment with BCP reagent (1-bromo-3-chloropropane, Molecular Research Center, OH, USA) to separate the RNA layer from DNA and protein. The extract was centrifuged (12000 × *g*, at 4°C for 15 minutes) and RNA was precipitated using isopropanol, washed twice (75% ethanol) and dried (vacuum centrifugation). The RNA pellet was re-suspended in 20μL of nuclease free water (Ambion) prior to PCR analysis. PCR was carried out on Applied Biosystems Step One RT-PCR machine with 48-well plate format. Triplicates from each sample of mouse tissue were analyzed, normalized to control values for Iba-1, CD68, and CD11c expression.

### Statistics

Statistical analyses were performed using one-way ANOVA to confirm overall significance (GraphPad Prism, v6.0, San Diego, CA). For comparisons between genotype (WT and CCR2-KO) and control and irradiated groups, parametric two-tailed unpaired t-tests were performed with Welch’s correction. All data are shown as the mean ± SEM of 4 independent observations.

## Results

### Effects of LDIR on newly born cell survival in the hippocampal DG in WT versus CCR2-KO animals

**Fig 2:**
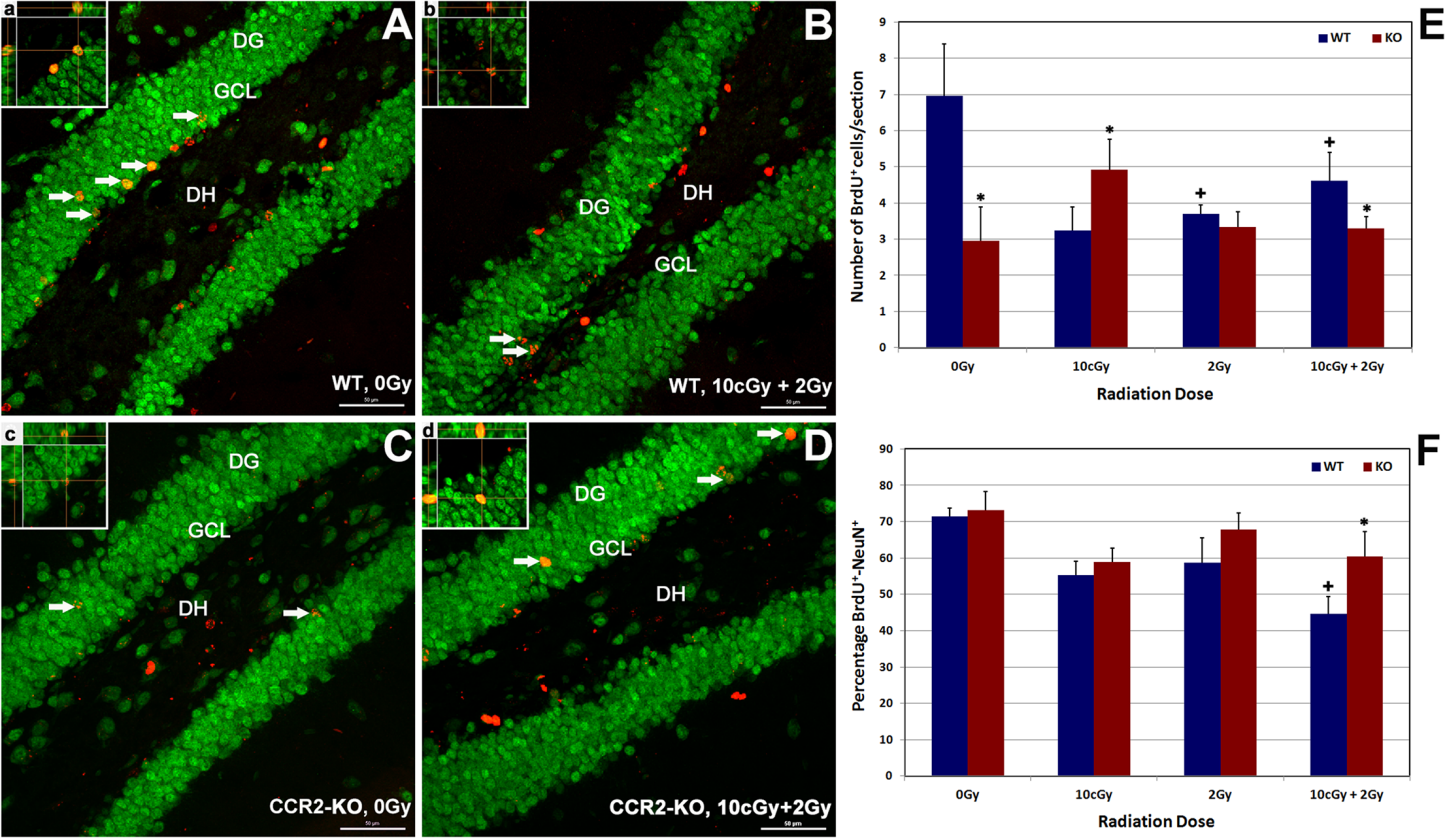
Effect of low dose ionizing radiation on hippocampal proliferation and neurogenesis within WT (A-B and a-b) and CCR2-KO (C-D and c-d) background. Dual immunofluorescence staining for BrdU positive cells (red) co-labeled with NeuN (green) was identified using laser scanning confocal microscopy (arrows). Exposure to low dose priming (10cGy) or challenge (2 Gy) irradiation lead to declined proliferation (BrdU positive cells) in the WT hippocampus significantly in comparison with CCR2-KO background (**E**) when analyzed 1 month post-irradiation. Formation and maturation of neurons (BrdU-NeuN) reduced significantly following 10 cGy + 2 Gy combination irradiation in the WT brain, whereas percentage of BrdU-NeuN was unchanged in the CCR2-KO hippocampus (**F**). Orthogonal reconstruction of 1 micro thick Z-stacks confirms presence of dual labeled cells (panel **a-d**). DG, dentate gyrus; DH, dentate hilus and GCL, granule cell layer. Scales bars: 50 μm (**A-D**) and, 10 μm (**a-d**). Data are shown as mean ± SEM of 4 independent observations. *, p = 0.05 indicates comparisons between WT and CCR2-KO genotypes. +, p = 0.05 indicates comparison with 0 Gy group.

To evaluate the effects of 10 cGy priming and 2 Gy challenge LDIR on the yield of proliferating cells surviving in the hippocampus 1-month following irradiation, the number of BrdU positive cells was quantified in WT and CCR2-KO mice (**Fig 2**). Immunoreactivity of BrdU (red) was located within the dentate SGZ and granule cell layer (GCL) of the hippocampus and was quantified as the number of BrdU positive cells per section (**Fig 2A–2D**and inserts, **a-d**). Overall ANOVA for the number of BrdU-positive cells was significant (p = 0.001) between the WT and CCR2-KO mice. Quantification of confocal micrographs indicated that compared to WT, CCR2 deficiency reduced significantly (48%, p = 0.05) the level of basal, newly born cell survival (0 Gy group) in the hippocampus (**Fig 2E**). Exposure of WT animals led to significant reductions (20–50%, p = 0.05) in the number of BrdU-positive cells 1-month post-irradiation, an effect that was relatively dose-independent under each of the dosing paradigms used (**Fig 2E**). In contrast, the numbers of BrdU-positive cells within the CCR2 deficient hippocampus were comparable to unirradiated controls (0 Gy) irrespective of the irradiation paradigm. Compared to 0 Gy controls, exposure of CCR2-KO animals to LDIR (10 cGy) led to a significant increase (60%, p = 0.05) in the number of surviving BrdU-positive cells (**Fig 2E**). Overall results showed that compared to WT animals, CCR2 deficiency protected animals against LDIR-induced reductions in cell survival, which may reflect changes in hippocampal proliferation.

### Effects of LDIR on hippocampal neurogenesis in WT versus CCR2-KO animals

To explore the effects of LDIR on neurogenesis within the WT and CCR2-KO brain, the percentage of BrdU-NeuN dual-labeled cells was quantified in each group (**Fig 2A–2D and 2F**). Dual immunofluorescence was identified by laser scanning confocal microscopy to quantify BrdU (red) and NeuN (green) staining within the hippocampal GCL (**Fig 2A–2D**). Overall ANOVA revealed a significant (p = 0.001) effect between WT and CCR2-KO animals for the percentage of BrdU-NeuN dual-stained cells. Basal neurogenesis (0 Gy) between each strain were not found to differ statistically (**Fig 2F**). Following exposure to either 10 cGy or 2 Gy, the percentage of BrdU-NeuN positive cells was not affected significantly in either strain of animal. However, differences in neurogenesis were found between the strains following the radioadaptive dosing paradigm. In WT mice, the percentage of dual-labeled cells declined significantly (37%, p = 0.05) following the priming and challenge doses compared to unirradiated controls (**Fig 2F)**. In contrast, the percentage of dual-labeled cells did not change in CCR2-KO mice exposed to radioadaptive dosing compared to unirradiated controls, but did show a significant increase in the percentage (15%, p = 0.05) of BrdU-NeuN positive cells compared to WT mice treated under the same conditions (**Fig 2F**). These data provide further evidence that neurogenesis following LDIR is relatively unaffected in CCR2 deficient animals.

### Quiescent microglia (Iba-1 positive) in WT and CCR2-KO animals following LDIR

**Fig 3:**
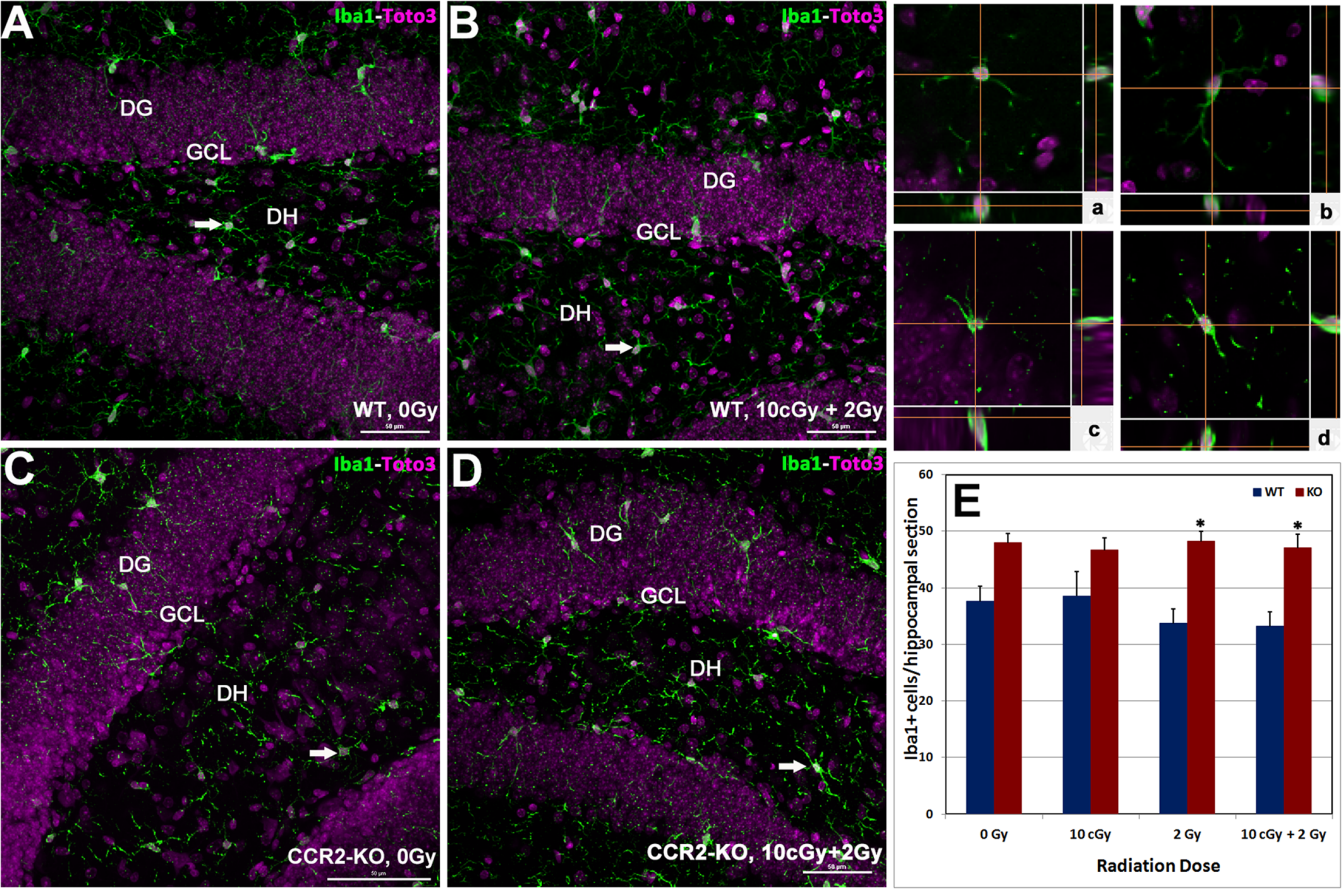
Status of hippocampal microglia following low dose ionizing radiation exposure. Immunofluorescence staining for Iba-1 positive (green), resting microglia, with nuclear counter staining (Toto-2, pink) was identified using laser scanning confocal microscopy (**A-D**). Iba-1 positive cells were distributed throughout the hippocampus (DG, DH, CA3 and CA1 subfields) and orthogonal Z-stacks confirmed the microglial morphology (panel **a-d)**. In general, numbers of Iba-1 positive cells were higher in CCR2-KO hippocampus comparison with WT animals (**E**). Following exposure to 2 Gy alone or 10 cGy + 2 Gy combined irradiation, the number of Iba-1 positive cells elevated significantly in the CCR2-KO mice compared to WT when analyzed 1 month post-irradiation. DG, dentate gyrus; DH, dentate hilus and GCL, granule cell layer. Scales bars: 50 μm (**A-D**) and, 10 μm (**a-d**). Data are shown as mean ± SEM of 4 independent observations. *, p = 0.05 indicates comparisons between WT and CCR2-KO genotypes.

To further understand the impact of LDIR on the inflammatory status of the hippocampus in WT and CCR2-KO mice, the number of Iba-1 positive resting microglia were quantified. Immunoreactivity of Iba-1 (green) was evident within each hippocampal subfield (DG, CA1 and CA3) and quantified as the number of Iba-1 positive cells per section (**Fig 3A–3D**and inserts, **a-d**). Overall ANOVA for the number of Iba-1 positive resting microglia was significant (p = 0.001) between the WT and CCR2-KO mice. Compared to WT, trends toward higher numbers of Iba-1 positive microglia were observed in unirradiated CCR2 deficient animals as well as those exposed to 10 cGy (**Fig 3E**). Cohorts from either genotype exposed to irradiation did not exhibit significant changes in the number of Iba-1 positive microglia in the hippocampus (**Fig 3E**).

### Gene expression status for pro-inflammatory markers following LDIR

**Fig 4:**
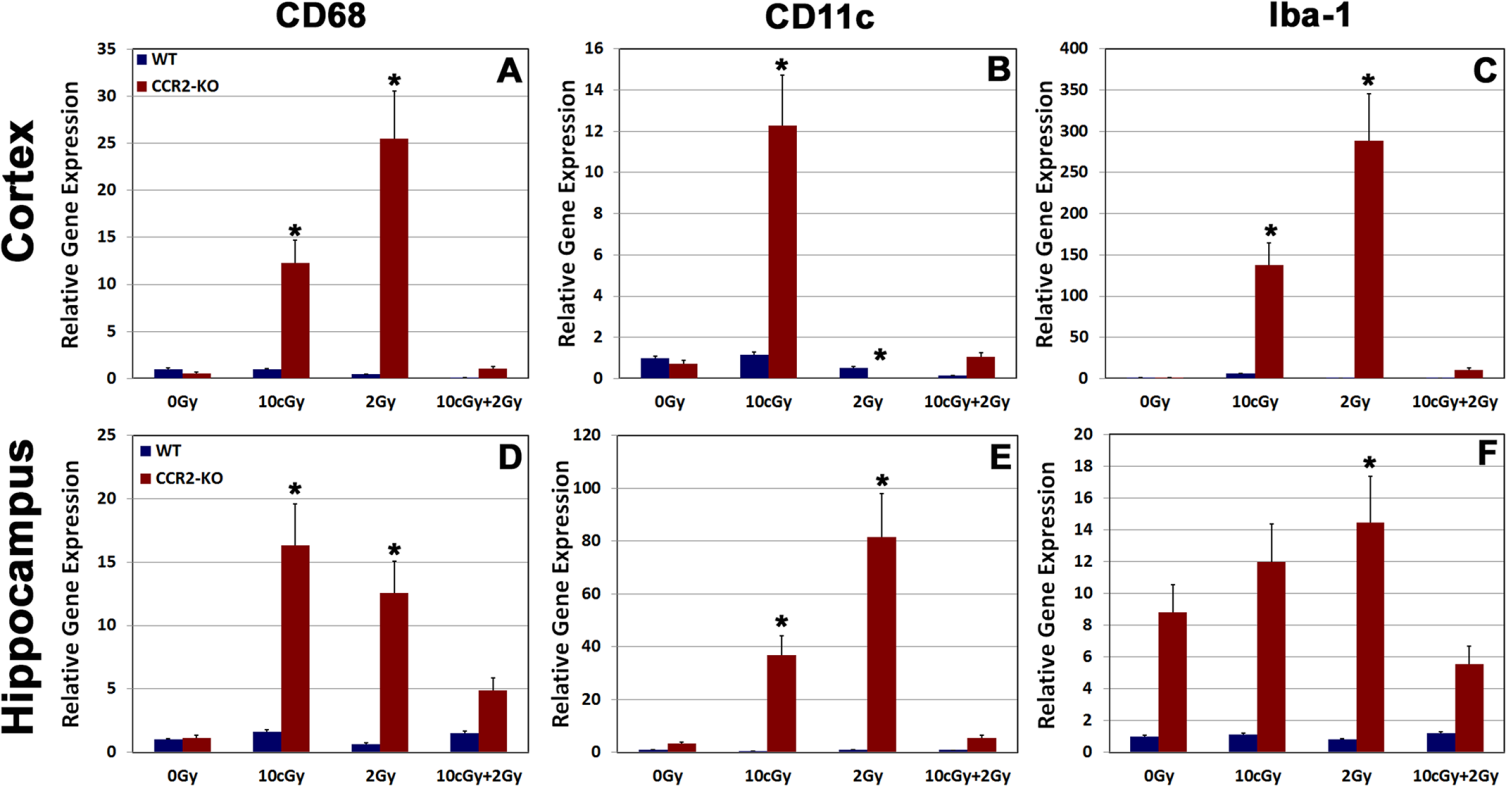
Gene expression analysis for pro-inflammatory markers from freshly dissected cortex and hippocampus. Relative gene expression for pro-inflammatory markers (CD68, CD11c and Iba-1) was analyzed using quantitative real-time PCR (qPCR) in cortex (**A-C**) and hippocampus (**D-F**) within the WT and CCR2-KO background. The mRNAs for all pro-inflammatory markers elevated significantly following 10 cGy or 2 Gy irradiation within the CCR2-KO animals (**A-F**). In contrast, relative gene expression for these pro-inflammatory markers drops down following 10 cGy + 2 Gy irradiation paradigm (**A-F**). WT animals did not display alterations in gene expression profile following irradiation. Data are shown as mean ± SEM of 4 independent observations. *, p = 0.05 in comparison with 0 Gy group.

To further analyze the effects of LDIR on expression of pro-inflammatory markers within the WT and CCR2-KO brain, we carried out quantitative RT-PCR for Iba-1, CD68 and CD11c from freshly dissected cortical and hippocampal tissues (**Fig 4**). RT-PCR data revealed that expression of Iba-1, CD68 and CD11c did not changed significantly in the WT brain following exposure to LDIR. In contrast, gene expression of all pro-inflammatory markers elevated significantly (p = 0.05) within the CCR2-KO brains when exposed to either single 10 cGy or 2 Gy radiation doses (**Fig 4**). The expression level of each pro-inflammatory marker was however, reduced significantly (p = 0.05) following radioadaptive dosing in CCR2-deficient animals (**Fig 4**).

## Discussion

Data from us and others have shown the importance of oxidative stress and neuroinflammation as important neurochemical mechanism regulating the functionality of the irradiated CNS [3, 6, 11, 17, 20]. Higher dose exposures more typical in the clinical setting have been shown to engage a variety of signaling pathways involving chemokines [8, 15, 17]. Alterations in chemokines receptors and/or ligands has been linked directly to neurodegenerative conditions and impaired cognition [22,23]. CCR2 receptors are constitutively expressed by microglia, NSCs and major neurons throughout the brain [18, 19] and altered chemokine signaling in response to tissue injury can impact the functionality of the CNS.

Our results on neurogenesis and microglia (**Figs 2**and **3**) show that CCR2 deficiency alters the cellular (NSC survival and/or proliferation, differentiation and Iba-1) and molecular (pro-inflammatory gene expression) response of the hippocampus following LDIR. While the number of surviving (BrdU positive) cells in the CCR2-KO compared to the WT hippocampus were low, acute or radioadaptive irradiation paradigms did not cause further reductions in survival in the CCR2 deficient background 1-month post-irradiation (**Fig 2E**). Moreover, the capability of forming new neurons (BrdU-NeuN positive cells) remained intact within the CCR2-KO hippocampus following exposure to the radioadaptive dosing paradigm (**Fig 2F**), an effect not observed in WT mice when analyzed one month post-irradiation. While our neurogenic measurements are limited in that they provided only relative rather than absolute quantification of differentiated phenotypes, results did corroborate past data demonstrating a neuroprotective role of CCR2 blockade [8, 15, 17]. Constitutive expression of CCR2 receptors by hippocampal NSCs and mature neurons [18, 19] makes them sensitive to radiation-induced changes in chemokine signaling. Thus, CCR2 deficiency may simply dampen the sensitivity of the CCR2 signaling axis to LDIR that manifests as a neuroprotective phenotype over protracted post-irradiation intervals.

Microglial activation helps modulate the innate immune response in a number of neurodegenerative conditions and during tissue remodeling following radiation injury [8, 17, 24–26]. In the CCR2 deficient background, data indicates that the number of resting microglia (Iba-1 positive) was relatively high in comparison to WT controls following each of the irradiation paradigms. Furthermore, the levels of resting microglia were not responsive to acute or radioadaptive dosing, as the numbers of Iba-1 positive cells remained constant over the 1-month post-irradiation interval (**Fig 3E**). Analysis of mRNA (qPCR) within the CCR2-KO brain indicated a significant increase in the message levels of pro-inflammatory markers (CD68, CD11c and Iba-1) following 10 cGy and 2 Gy acute exposures in the hippocampus and neocortex (**Fig 4**). Interestingly, elevated proinflammatory markers after acute exposure were attenuated significantly in the brains of CCR2 deficient mice after radioadaptive dosing (**Fig 4**). Exposure to low priming doses of radiation (10 cGy) may reset the inflammatory tone within the CCR2-KO hippocampal microenvironment, thereby preventing the upregulation of inflammatory markers following subsequent exposure to higher (2 Gy) challenge doses (**Fig 4**). In sum, our findings demonstrate that the low dose radiation response between WT and CCR2 deficient mice is different, possibly mediated by a CCR2 dependent signaling feedback.

Radiation exposure of the brain disrupts neurotransmission and elicits varying degrees of cognitive dysfunction. Multiple mechanisms are involved, including the inhibition of hippocampal neurogenesis [6], the degradation of neuronal structure and the alteration of synaptic integrity and neuronal plasticity [3, 11, 27]. Irradiation of the brain also triggers a complex temporal response of secondary reactive processes involving oxidative stress and inflammation [2, 4–6, 10]. Changes in redox metabolism promote early and recurrent inflammatory signatures that perturb the neurogenic microenvironment of the hippocampus. Pharmacologic modulation of inflammatory and oxidative signaling via peroxisome proliferating-activated receptor (PPAR) agonists has been shown to be neuroprotective following whole-brain irradiation [28,29]. Past and current studies have now demonstrated that genetic disruption of chemokine signaling can alter the radioresponse of the CNS, mediated in part through changes in the levels of microglia [8, 17]. Importantly, our quantitative measurements of neurogenesis and microglia corroborate past data collected at higher doses, and confirm that similar neuroprotective phenotypes transpire after LDIR. Our data supports the potential utility of attenuating chemokine signaling to reduce CNS damage following acute and/or protracted LDIR exposure encountered under a variety of occupational and medical scenarios.
